# Cardiac Output and Performance during a Marathon Race in Middle-Aged Recreational Runners

**DOI:** 10.1100/2012/810859

**Published:** 2012-05-03

**Authors:** Véronique L. Billat, Hélène Petot, Morgan Landrain, Renaud Meilland, Jean Pierre Koralsztein, Laurence Mille-Hamard

**Affiliations:** ^1^UBIAE, U902 INSERM, University of Evry-Val-D'Essonne, 91025 Evry, France; ^2^Sports Medicine Center, CCAS, Paris, France

## Abstract

*Purpose*. Despite the increasing popularity of marathon running, there are no data on the responses of stroke volume (SV) and cardiac output (CO) to exercise in this context. We sought to establish whether marathon performance is associated with the ability to sustain high fractional use of maximal SV and CO (i.e, cardiac endurance) and/or CO, per meter (i.e., cardiac cost). *Methods*. We measured the SV, heart rate (HR), CO, and running speed of 14 recreational runners in an incremental, maximal laboratory test and then during a real marathon race (mean performance: 3 hr 30 min ± 45 min). *Results*. Our data revealed that HR, SV and CO were all in a high but submaximal steady state during the marathon (87.0 ± 1.6%, 77.2 ± 2.6%, and 68.7 ± 2.8% of maximal values, respectively). Marathon performance was inversely correlated with an upward drift in the CO/speed ratio (mL of CO × m^−1^) (*r* = −0.65, *P* < 0.01) and positively correlated with the runner's ability to complete the race at a high percentage of the speed at maximal SV (*r* = 0.83, *P* < 0.0002). *Conclusion*. Our results showed that marathon performance is inversely correlated with cardiac cost and positively correlated with cardiac endurance. The CO response could be a benchmark for race performance in recreational marathon runners.

## 1. Introduction

Endurance running capacity may have initially arisen in the genus *Homo* [1, 2]. Over the course of evolution, human physiology has been optimized for covering large distances every day, in order to find enough food to sustain the brain's metabolism. Indeed, the increasing popularity of marathon running in modern humans of all ages and abilities can be viewed as a legacy of our species' evolutionary capacity to run long distances (>5 km) using aerobic metabolism [2]. Indeed, the number of starters in the London Marathon has risen from 7,000 to 35,000 over the last 30 years and participation in road racing in general has increased by more than 50% over the last decade [3–5]. The increasing popularity of road running is typified by the emergence of recreational marathon runners who complete the 42.195 km event in a time of between 2 hr 40 min and 4 hr 40 min. The marathon's potentially negative impact on cardiac status and the occurrence of sudden cardiac deaths during this type of event have prompted much debate [3].

Along with ${\dot{\text{V}}\text{O}}_{2\max}$ and energy cost, endurance (i.e., the ability to sustain a high fraction of$\;{\dot{\text{V}}\text{O}}_{2\max}$) is one of the three benchmarks for overall performance in marathon running [6]. Indeed, high-level runners display high fractional use of their maximal oxygen uptake (${\dot{\text{V}}\text{O}}_{2\max}$), with peak recorded values of 88% [6, 7]. In two recreational runners, Maron et al. used direct measurements to show that the fractional use of ${\dot{\text{V}}\text{O}}_{2\max}$ ranged between 68% and 100% at various points in a free-pace marathon race [8]. These data suggest that cardiac strain is quite high [6, 9–11]. Hence, the question of physiological strain also arises when considering the emerging class of middle-aged, recreational marathon runners; the evaluation of cardiac strain and cardiac responses during a marathon is, therefore, a topic of legitimate interest. Other studies have reported a progressive increase in fractional use of the maximum heart rate (HR_max⁡_) over the course of the race (from around 80% of HR_max⁡_ at the start to around 90% at the finish) [12–14]. This HR increase was associated with a continuous speed decrease, starting halfway through the race (i.e., at 21 km). The upward drift in HR is one component of so-called “cardiovascular drift,” which is also characterized by a decrease in stroke volume (SV) and in arterial and pulmonary pressures. Depending on the exercise intensity, cardiac output (CO) may or may not be maintained over time according to the [15]. However, to the best of our knowledge, no data on the CO response during a marathon race are available.

An increasing number of recreational marathon runners are now using data from HR and speed monitors in an attempt to pace their effort. There are currently no guidelines on how to use these variables to optimize performance [16]. Furthermore, there is still debate as to limiting factors in marathon racing in general and cardiac limitation in particular [ 17, 18, 19]. Hence, we continuously measured SV, HR, CO, and speed over the course of a marathon race and sought to establish whether marathon performance (and notably the speed decrease typically seen over the second half of the race) is associated with the ability to sustain high fractional use of the maximal SV and CO (i.e., cardiac endurance) and/or with the CO per meter (i.e., cardiac cost). 

## 2. Methods

### 2.1. Subjects

We investigated performance in 14 middle-aged, male, recreational marathon runners (all of whom were national-level short-distance triathletes or international-level cyclists). The subjects were free of known cardiac and pulmonary diseases. The study population's physical characteristics (mean ± SD age: 37 ± 6  years; weight: 71 ± 8 kg; height 178 ± 6 cm) are summarized in Table 1. Each subject was familiarized with the experimental procedures prior to the study measurements. Before participation, subjects received a verbal explanation of the nature of the study (including the risks associated with performing a maximal physical effort) and voluntarily gave their written, informed consent. The present study complied with the ethical standards set by the Declaration of Helsinki and all study procedures were approved by the local investigational review board.

#### 2.1.1. Experimental Design

Two weeks before participation in the Paris marathon, the subjects performed a laboratory-based incremental test on a treadmill. This test was performed until exhaustion and enabled us to record the maximal values of a number of physiological parameters. The laboratory-based protocol also enabled the subjects to familiarize themselves with the material to be worn on race day.

#### 2.1.2. Laboratory Tests

**Protocol**

After familiarization with the laboratory and the test procedures, the subjects each performed a conventional, incremental running test on a treadmill (h/p/cosmos, Nussdorf-Traunstein, Germany) until volitional exhaustion. The running speed was set to 8 km/h and then increased by 1 km/h every 3 min until exhaustion. The subjects were given verbal encouragement during the test. We determined the maximal oxygen uptake (v$\dot{\text{V}}$O_2max_), maximal HR (HR_max_), maximal SV (SV_max_), speed at SV_max_ (vSV_max)_ [17, 20], and speed at the lactate threshold (vLT).

#### 2.1.3. Data Collection Procedures

**Measurement of Gas Exchanges during the Test**

Oxygen uptake was measured breath-by-breath using a Quark b^2^ (Cosmed, Rome, Italy). Expired gas concentrations were averaged every 5 s. Before each test, the Quark b^2^ was calibrated according to the manufacturer's instructions. The turbine flow-meter was calibrated using a 3L syringe (Quinton Instruments, Seattle, WA) [21].

**Blood Lactate Measurements**

Capillary blood was sampled from a fingertip and assayed for lactate (Lactate Pro LT, Arkay Inc., Kyoto, Japan) [22]). For the test, samples were taken at rest, at the end of each stage, at the end of the test, and after two and four minutes of posttest recovery. The $\dot{\text{V}}{\text{O}}_{2}$ at the LT was defined as the starting point of a rapid lactate accumulation of *∼*4 mM and was expressed as a percentage of $\dot{\text{V}}{\text{O}}_{2\max}$ [23]. For the marathon, blood lactate was measured at the start of the race and two minutes after the finish.

**Cardiovascular Measurements**

An impedance cardiography device (PhysioFlowType PF05L1, Manatec, Macheren, France) was used to determine HR, SV, and CO during the test and during the marathon. This device is exactly the same as the manufacturer's PhysioFlow Lab1 system but had been miniaturized for the purposes of the present study. The theoretical basis for this technique and its application to and validity in exercise testing have been described by others [24–28] and in our laboratory [29].

To calculate SV, the PhysioFlow measures changes in transthoracic impedance (dZ) during cardiac ejection. The PhysioFlow emits high-frequency (75 kHz) and low-amperage (3.8 mA peak-to-peak) alternating electrical current via skin electrodes [19]. Two pairs of electrodes (a pair of transmitters and a pair of receivers) are applied one above the others (so as to not overlap) at the supraclavicular fossa at the left base of the neck and at the midpoint of the thoracic spine region. An additional pair of electrodes is used to monitor a single electrocardiogram (ECG) lead placed in the V1/V6 position. After entry of patient data (including the resting systolic and diastolic blood pressure values), the resting stroke volume index (SV_ical_, mL·m^−2^) is initially evaluated during an auto-calibration procedure for 30 consecutive heartbeats recorded in a seated, upright position. The auto-calibration stores the largest impedance variation during systole (*Z*peak−*Z*min) and the largest rate of variation of the impedance signal (the contractility index, *dZ*/*dt*peak). The magnitude of the SVi depends on the thoracic flow inversion time (TFIT, m·s^−1^), measured from the first derivative of the impedance signal. In fact, TFIT is the time interval between the first zero value after the beginning of the cardiac cycle (the start of the QRS complex on the ECG) and the first trough after the peak ejection velocity (*dZ*/*dt*peak). During data acquisition, variations in these parameters are analyzed and compared with those obtained during calibration. The parameter SV_ical_ is calculated according to the following formula:

(1) $${\text{SV}}_{\text{ical}}\;=\;k\times \left[\frac{\left(dZ/dt\max\right)}{\left(Z\max-Z\min\right)}\right]\times W\left(\text{TFITcal}\right),$$

where *k* is an empirically adjusted constant and *W* is a proprietary correction algorithm. Each displayed SV represents the mean value over a 15 s, artifact-free period [19, 30]. The device calculates CO (in L·min^−1^) according to the following formula:

(2) CO=  HR×SVi×BSA,

where HR is based on the R-R interval in the first derivative of the ECG signal (dECQ/*t*, which provides a more stable signal than the ECG itself), SVi is determined as above, and the body surface area (BSA) is the calculated according to Haycock's equation:

(3) BSA  =0.024265×BM0.5378×H0.3964,

where BM is the body mass in kg and H is the height in cm. According to the manufacturer, the PhysioFlow is novel because it calculates SV independently of baseline impedance (*Z*_0_). The latter causes many problems in conventional approaches to measuring bioimpedance because its value is greatly affected by hydration status, the inter-electrode distance, and the resistivity of the blood. To avoid this problematic variable, PhysioFlow does not measure *Z*_0_ during or after calibration and relies only on *dZ*. This is important in the current investigation, since where exercise might be expected to change the pulmonary capillary blood volume. However, the changes in baseline thoracic *Z*_0_ caused by fluid expansion in the lungs should not disturb the measurement (unless they have a true hemodynamic impact which modifies the pulsatile waveform morphology). The PhysioFlow has been previously validated against the direct Fick method. Mean differences between CO values obtained using the direct Fick method and the PhysioFlow device are not significant during rest (0.04 L·min^−1^) [19], submaximal exercise (0.29 L·min^−1^) [19], or maximal incremental exercise (0.58 L·min^−1^) [19]. The direct Fick method is also highly correlated with the PhysioFlow results during rest (*r* = 0.89, *P* < 0.001, *n* = 40) [19], submaximal exercise (*r* = 0.85, *P* < 0.001, *n* = 40) [30], and maximal exercise (*r* = 0.94, *P* < 0.01, *n* = 50) [30]. Strong correlations between the direct Fick method and impedance cardiography have also been reported for SV (*r* = 0.84, *P* < 0.001) and CO (*r* = 0.98, *P* < 0.001) during maximal cycling exercise in young, fit men [31].

The SV and HR were measured continuously during each test, with beat-to-beat smoothing via 5 s averaging algorithm. Before each test, the PhysioFlow was calibrated according to the manufacturer's instructions. Cardiac output was subsequently calculated using standard equations.

#### 2.1.4. Cardiovascular Data Analysis

A $\dot{\text{V}}{\text{O}}_{2\max}$ plateau was identified if the $\dot{\text{V}}$O_2_ value (in mL·kg^−1^·min^−1^) for a given power level was less than 1.75 times that measured for the previous power level. If no $\dot{\text{V}}{\text{O}}_{2\max}$ plateau was observed, the attainment of $\dot{\text{V}}{\text{O}}_{2\max}$ was confirmed by the following secondary criteria: (a) a respiratory exchange ratio greater than 1.10, (b) an HR > 95% of the theoretical age-predicted maximum, (c) a rating of perceived exertion > 16, and (d) a blood lactate concentration above 8 mM.v$\dot{\text{V}}{\text{O}}_{2\max}$ was defined as the lowest velocity that elicited $\dot{\text{V}}{\text{O}}_{2\max}$ [20]. If a subject achieved $\dot{\text{V}}{\text{O}}_{2\max}$ during the last stage but was unable to complete the full 3 minutes, v$\dot{\text{V}}{\text{O}}_{2\max}$ was calculated as

(4) $${\text{v}\dot{\text{V}}\text{O}}_{2\max}=\text{vF}+\left[\left(\frac{t}{180}\right)\times 1\right],$$

where first the speed at the last complete stage (m·s^−1^), *t, * is the duration over which the last workload was maintained (s) and 1 is the speed increment (km·h^−1^) between the last two stages.

**Anthropometry**

Height and weight were measured before and after each test. Five skin-fold measurements were made (triceps, biceps, supra-iliac, subscapular, and mid-thigh) and the percentage body fat was estimated according to the Durnin-Womersley method [32].

#### 2.1.5. Marathon Data Measurement and Analysis

Speed (V) was measured using an accelerometer (the RS800 CX from Polar Electro Oy, Oulu, Finland) [33]. Heart rate, SV, and CO were indexed according to running speed (HRS, SVS, and COS, respectively, with the following units: beats per meter for HRS, blood volume per beat per meter run for SVS, and blood volume per meter run for COS). The COS parameter was considered as an index of cardiac cost by analogy with oxygen cost (i.e., the oxygen uptake per meter run).

#### 2.1.6. Statistical Analysis

Descriptive statistics are quoted as the mean and standard deviation (SD). The normality of the data distribution was checked with a Fisher-Snedecor test. An analysis of variance (ANOVA) for repeated measures and a Scheffe *posthoc* test were applied to the V, HR, SV, CO, HRS, SVS, and COS data for every 10% of the race distance (i.e., every 4 km). Furthermore, a Student's *t*-test for paired data was used to compare the cardiac responses measured during the marathon and during the incremental test. For each individual, the slope of the plots of HRS, SVS, and COS against performance (i.e., the average marathon speed) were assessed using least-squares linear regression. The threshold for statistical significance was set to *P* < 0.05.

## 3. Results

### 3.1. Fractional Use of SV, HR, CO, and Speed during the Marathon Race

**Figure 1:**
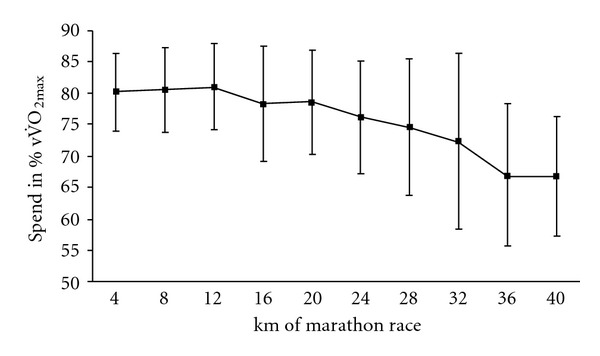
running speed (as a percentage of speed at $\dot{\text{V}}{\text{O}}_{2\max}$) decreases during the marathon.

Before considering the fractional use of maximal cardiac parameters, it must be borne in mind that the average marathon speed (vMar) in this group of recreational marathon runners group was submaximal (73.8 ± 8.8% of v$\dot{\text{V}}{\text{O}}_{2\max}$, on average) (Figure 1). This value was significantly lower than vSVmax (80.5 ± 6.1% v$\dot{\text{V}}{\text{O}}_{2\max}$) and vLT (79.7 ± 4.8%  $\dot{\text{V}}{\text{O}}_{2\max}$) measured during the incremental test (Tables 1 and 2). Fractional use of SV_max_, HR_max_, and CO_max_ during the race is summarized in Table 1. On average, the marathon race elicited 68 ± 2% of SV_max_, 87 ± 2% of HR_max_, and 77 ± 3% of CO_max_ measured in the incremental test.

**Figure 2:**
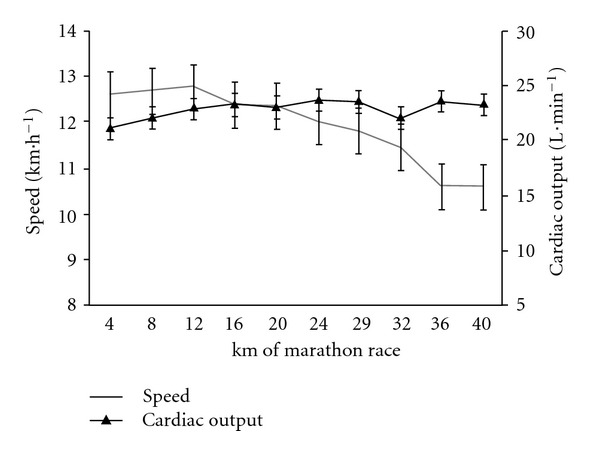
Speed (km·h^−1^, grey line) and CO (L·min^−1^, black line) during the marathon.

**Figure 3:**
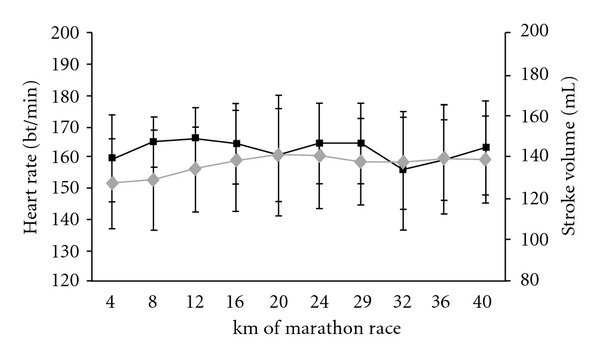
Heart rate (beats·m^−1^, black line) and stroke volume (mL, grey line) during the marathon.

**Figure 4:**
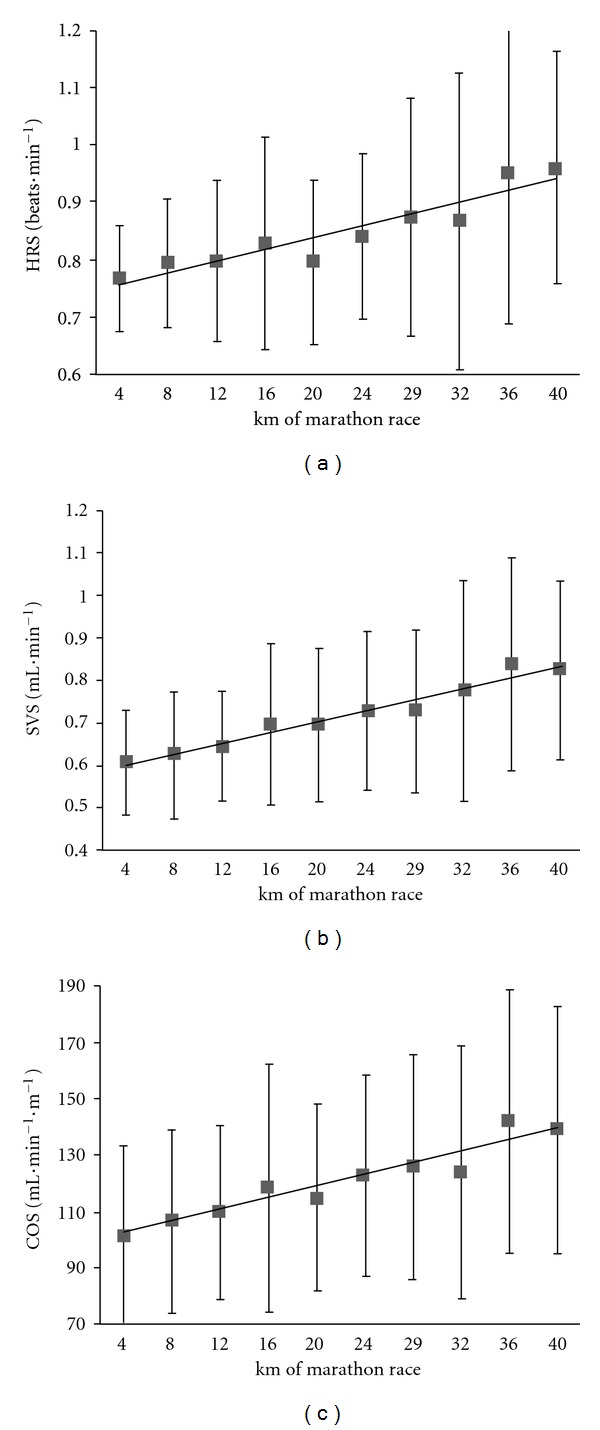
Increases in the heart rate per meter run (HRS, panel (a)), stroke volume per meter run (SVS, panel (b)), and blood flow per meter run (COS, panel (c)) during the marathon race.

The cardiac variables, CO, SV, and HR, did not differ significantly when comparing the first 4 km and last 4 km of the race (*P* = 0.2 for CO and SV; *P* = 0.5 for HR; *t*-test for paired data) (Figures 2 and 3). However, significant variations in HR over the course of the marathon were revealed by an ANOVA with repeated measures. Indeed, the HR increased until km 12, stabilized until km 28 and then decreased to the finish (*F* = 3.0, *P* = 0.003) (Figure 3). However, this stabilization in cardiac parameters must be viewed in light of the mean 18 ± 9% decrease in running speed between km 12 and the finish (*P* < 0.0001). Accordingly, the values per meter run were significantly greater for km 36–40 than for km 8–12 (Table 3), with increases of 21 ± 16% for HRS (Figure 4(a)), 25 ± 17% for SVS (Figure 4(b)), and 26 ± 22% for COS (Figure 4(c)). The COS increase was caused by upward drifts in HRS (*r* = 0.69, *P* = 0.005) and SVS (*r* = 0.85, *P* < 0.0001) but not by the speed decrease (*r* = −0.23, *P* = 0.42). In contrast, the increase in HRS was highly correlated with the speed decrease between km 8–12 and km 36–40.

### 3.2. Marathon Performance Correlates Negatively with the Upward Drift in the COS (Cardiac Cost) but Positively with the Ability to Sustain High Fractional Use of the Maximal SV and CO (Cardiac Endurance)

**Figure 5:**
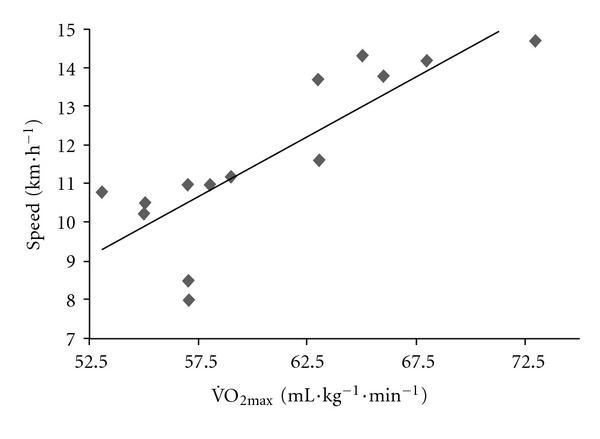
The relationship between mean speed in the marathon (km·h^−1^) and $\dot{\text{V}}{\text{O}}_{2\max}$ (mL·kg^−1^·min^−1^) at v_Mar_.

**Figure 6:**
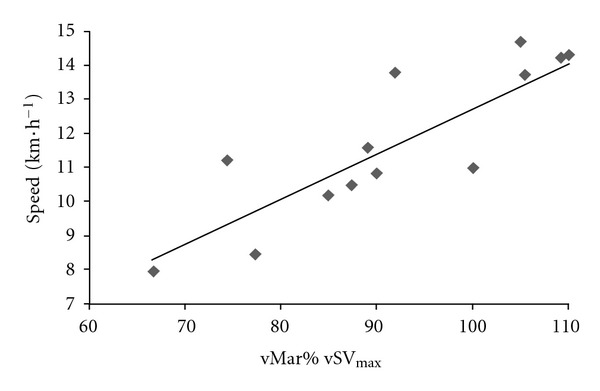
The relationship between mean speed in the marathon (km·h^−1^) and vMar_%_vSV_max_.

**Figure 7:**
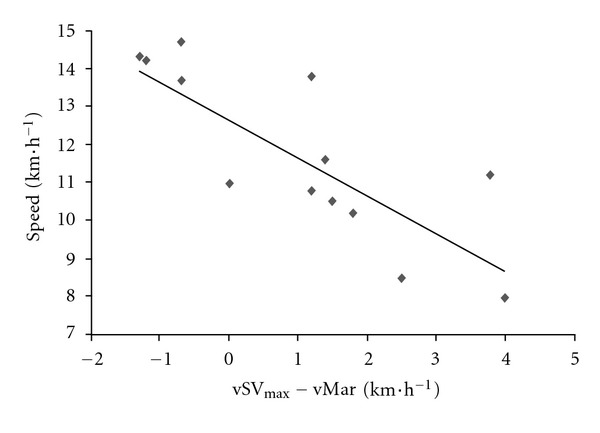
The relationship between mean speed in the marathon (km·h^−1^) and the difference between vSV_max_ and vMar (km·h^−1^).

In contrast to what is often believed, the speed decrease was not correlated with overall performance; the fastest runners overall were not those who had the smallest relative speed decrease (as a percentage of the speed at km 12) (*r* = 0.39, *P* = 0.16). However, the present study confirmed that as typically reported, marathon performance was strongly correlated with $\dot{\text{V}}$O_2max_ (*r* = 0.83, *P* < 0.0002, Figure 5) and the fractional use of $\dot{\text{V}}$O_2max_ at vLT (*r* = 0.59, *P* = 0.03) but not with the fractional use of $\dot{\text{V}}$O_2max_ at the maximal stroke volume reached in the incremental test (84.9 ± 10.5%) (*P* = 0.65). The cardiac response during the incremental test was not correlated with marathon performance; the cardiac response during the race itself was the marathon performance factor. Indeed, the fractional use of SV_max_ and of vSV_max_ during the marathon was moderately and strongly correlated with performance (*r* = 0.67, *P* = 0.03 and *r* = 0.83, *P* = 0.0002), respectively (Figure 6). This finding suggests that the best recreational marathon runners are those who (i) can sustain a high fraction of their vSV_max_ and (ii) have the lowest absolute difference between vSV_max_ and vMar (*r* = −0.78, *P* = 0.0009) (Figure 7).

**Figure 8:**
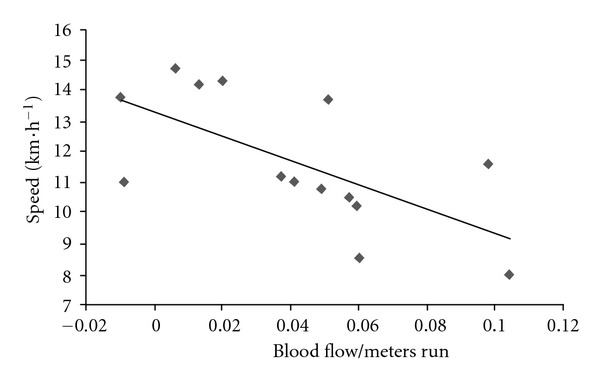
The relationship between mean speed in the marathon (km·h^−1^) and the drift in blood flow per meter run (COS) during the race (mL·m^−1^).

The relationship between our cardiac marathon performance indicators and conventional indicators is shown by the observation that the sustained fractional use of v$\dot{\text{V}}$O_2max_ during the marathon was strongly and positively correlated with the fractional use of vSV_max_ (*r* = 0.86, *P* < 0.0001) and inversely correlated with the upward drift in the CO/speed ratio (mL of CO × m^−1^) (*r* = −0.65, *P* < 0.01) (Figure 8).

## 4. Discussion

The present study is the first to evidence CO strain during a real race. Our results suggest that endurance performance in marathon running can most usefully be measured during the race itself by applying novel techniques for cardiac data collection. By applying an in-the-field approach, we found that marathon performance by middle-aged, recreational runners was associated with the ability to sustain high fractional use of the maximal SV and CO (i.e., cardiac endurance) and/or the CO per meter (i.e., cardiac cost).

Crandall and Gonzaléz-Alonso [26] have reported that a drop in SV may be a major limiting factor in exhaustive exercise [34]. However, we showed here that SV did not decrease over time; this agrees with previous reports of an SV steady state during large reductions in brain perfusion in the heat-stressed human and during intense, endurance exercise [26]. Indeed, SV remained at submaximal steady state (77 ± 3%), as did CO (69 ± 3%). Furthermore, an increase in HR in a neutral environment has been shown to be responsible for the SV decline in steady exercise performed for 1 hour at 57% of $\dot{\text{V}}$O_2max_ [35]. Therefore, in the present study, it was important to check whether SV declined and HR increased in a longer, more intense bout of exercise like the marathon. In the marathon studied here, we did not observe the cardiac drift that may be responsible for an increase in cardiac oxygen uptake (as estimated by the double product) during constant-load exercise [26]. Indeed, the runners' mean HR remained at a high, steady state throughout the race (87 ± 2% of HR_max_).

To the best of our knowledge, the hypothesis where by cardiac limitation is a marathon performance factor has not previously been evaluated directly. However, our direct, intrarace measurements showed that cardiac strain (CO, SV, and HR) remained in a submaximal, steady state; this was probably due to the decrease in running speed after km 12. However, this speed drop was not correlated with final performance; the best runners were not those who reported the lowest relative speed decrease between the first four km of the race and last four km.

As a result of the speed decrease and a steady state for the cardiac variables, the values per meter run (COS, SVS, and HRS) increased after km 12. Interestingly, the amplitude of the increase in COS (cardiac cost) between km 12 and the end race was strongly correlated with marathon performance (i.e., finishing time or average speed) and could be an important performance predictor. This cardiac cost increase could be related to the energy cost, which has been shown (in treadmill measurements) to be greater just after a marathon [6].

Lastly, our results prompted us to hypothesize that cardiac strain is both a pace maker and a consequence of the decrease in racing speed after km 12 (i.e., after about 90 minutes of running, on average). The speed decrease is thought to be due to glycogen and metabolic limitations, as reported by Rapoport [36]. Indeed, the latter author demonstrated that glycogen storage capacity was only a performance-limiting factor in runners with low or moderate aerobic capacities ($\dot{\text{V}}$O_2max_ < 60 mL·kg^−1^·min^−1^) or with relatively small leg muscles. The performance levels of the recreational marathon runners studied here are in accordance with Rapoport's predictions [36] on the basis of the percentage HR_max_, the $\dot{\text{V}}$O_2max_, and the LT. Therefore, the limitation on marathon performance must also include running economy. The latter depends on the cardiac cost, which in turn depends on the HR and thus the number of heart beats per km. Hence, these two hypotheses are not mutually exclusive, since the fractional use of SV, HR, and CO are not performance factors in marathon racing.

## 5. Conclusion

Our results showed that marathon performance is inversely correlated with an upward drift in the CO per meter ran (i.e., cardiac cost) and positively correlated with the ability to sustain high fractional use of the maximal SV and CO (i.e., cardiac endurance). Therefore, the CO response during the race could be a performance benchmark in recreational marathon runners. The present study of intersubject differences in performance factors is not a sufficiently methodological approach for explaining intrasubject marathon performance limitations. This type of work would require further intramarathon measurements (such as oxygen uptake and integrated electromyography) that are now becoming accessible with portable systems.

## Figures and Tables

**Table 1:** The subjects' characteristics and values during the incremental test.

Runner	Characteristics		Values during the incremental test					
	Age	BMI	SV_max_	HR_max_	CO_max_	vLT	vSV_max_	v_peak_
1	27	18.3	147	189	25.6	12	11	15
2	30	24.0	180	191	26.1	10	11	14
3	39	24.2	182	200	26.7	11	12	15
4	36	23.3	174	192	28.0	14	15	17
5	39	21.6	197	189	33.7	15	14	18
6	36	22.5	179	199	29.9	12	11	15
7	34	20.8	163	187	25.5	15	13	17
8	37	23.2	198	190	35.4	13	13	17
9	45	22.1	185	179	23.3	12	13	16
10	47	21.0	166	190	27.4	15	13	19
11	33	23.4	175	181	29.5	12	15	16
12	36	23.6	180	187	22.6	12	12	16
13	35	24.6	180	186	27.4	11	12	16
14	46	20.8	163	180	24.3	12	12	14

Mean	37	22.4	176	189	27.5	13	13	16
SD	6	1.7	14	6	3.7	2	1	1

Age (yrs); BMI: body mass index; SV_max_: maximal stroke volume (mL); HR_max_: maximal heart rate (beats·min^−1^); CO_max_: maximal cardiac output (L·min^−1^); vLT: speed at the lactate threshold (km·h^−1^); vSV_max_: speed at the maximal stroke volume (km·h^−1^); v_peak_: maximal speed (km·h^−1^).

**Table 2:** Performance during the marathon race.

Runner	T_mar_	vMar	vMar_%_vSV_max_	vMar_%_v$\dot{\text{V}}$O_2max_
1	3.51	11.0	100.0	77.3
2	4.58	08.5	77.3	60.7
3	4.08	10.2	85.0	72.9
4	3.04	13.8	92.0	81.2
5	2.52	14.7	105.0	81.7
6	3.51	11.0	100.0	73.3
7	2.58	14.2	109.2	83.5
8	3.05	13.7	105.4	85.6
9	3.39	11.6	89.2	72.5
10	2.57	14.3	110.0	79.4
11	3.45	11.2	74.4	70.0
12	5.10	08.0	66.7	53.3
13	4.02	10.5	87.5	70.0
14	3.54	10.8	90.0	77.1

Mean	3.50	11.7	92.3	73.9
SD	0.76	2.1	12.9	8.8

T_mar_: marathon time (hours); vMar: mean speed for the marathon (km·h^−1^), vMar_%_vSV_max_: mean marathon speed as a percentage of the speed at maximal stroke volume during the incremental test; vMar_%_v$\dot{\text{V}}$O_2max_: mean marathon speed as a percentage of the speed at maximal oxygen uptake during the incremental test.

**Table 3:** SV, HR, CO and speed (v%vMar) every 4 km of the marathon race, all expressed as a percentage of the maximal values in the incremental test.

Race km	SV% SV_max_	HR% HR_max_	CO% CO_max_	v%v$\dot{\text{V}}{\text{O}}_{2\max}$
4	72.2 ± 11.3	84.6 ± 7.4	76.6 ± 23.0	81.1 ± 9.4
8	73.0 ± 11.8	87.5 ± 4.2	80.6 ± 23.4	81.3 ± 7.5
12	76.0 ± 9.6	89.1 ± 4.0	85.0 ± 23.1	81.5 ± 6.4
16	78.4 ± 11.9	88.8 ± 3.8	85.0 ± 23.1	78.6 ± 8.1
20	79.4 ± 12.7	88.4 ± 3.1	83.5 ± 17.2	79.0 ± 8.4
24	79.4 ± 11.0	88.1 ± 4.9	86.1 ± 16.8	76.6 ± 9.0
28	78.0 ± 8.8	88.3 ± 4.9	85.9 ± 18.2	74.4 ± 5.2
32	77.6 ± 10.3	85.0 ± 7.3	82.6 ± 18.4	71.8 ± 8.9
36	78.8 ± 12.4	85.4 ± 7.5	82.9 ± 15.8	66.6 ± 6.2
40	78.7 ± 9.8	86.3 ± 8.5	84.7 ± 18.4	67.0 ± 7.5

Mean	77.2 ± 11.0	87.2 ± 5.6	83.3 ± 19.7	75.7 ± 7.7

Values are presented as the mean ± SD.

SV: stroke volume; HR: heart rate; CO: cardiac output; v%v$\dot{\text{V}}{\text{O}}_{2\max}$: the racing speed as a percentage of the speed at $\dot{\text{V}}{\text{O}}_{2\max}$ recorded in the incremental test.
